# Automation vs. Experience: Measuring Alzheimer’s Beta-Amyloid 1–42 Peptide in the CSF

**DOI:** 10.3389/fnagi.2018.00253

**Published:** 2018-08-22

**Authors:** Alexander L. Kollhoff, Jennifer C. Howell, William T. Hu

**Affiliations:** Department of Neurology, Emory University, Atlanta, GA, United States

**Keywords:** Alzheimer’s disease, biomarkers, cerebrospinal fluid, mild cognitive impairment, intermediate precision

## Abstract

Cerebrospinal fluid (CSF) biomarkers can enhance the early and accurate etiologic detection of Alzheimer’s disease (AD) even when symptoms are very mild, but are not yet widely available for clinical testing. There are a number of reasons for this, including the need for an experienced operator, the use of instruments mostly reserved for research, and low cost-effectiveness when patient samples do not completely fill each assay plate. Newer technology can overcome some of these issues through automated assays of a single patient sample on existing clinical laboratory platforms, but it is not known how these newer automated assays compare with previous research-based measurements. This is a critical issue in the clinical translation of CSF AD biomarkers because most cohort and clinicopathologic studies have been analyzed on older assays. To determine the correlation of CSF beta-amyloid 1–42 (Aβ42) measures derived from the automated chemiluminescent enzyme immunoassay (CLEIA, on Lumipulse^®^ G1200), a bead-based Luminex immunoassay, and a plate-based enzyme-linked immunoassay enzyme-linked immunosorbent assay (ELISA), we analyzed 30 CSF samples weekly on each platforms over 3 weeks. We found that, while CSF Aβ42 levels were numerically closer between CLEIA and ELISA measurements, levels differed between all three assays. CLEIA-based measures correlated linearly with the two other assays in the low and intermediate Aβ42 concentrations, while there was a linear correlation between Luminex assay and ELISA throughout all concentrations. For repeatability, the average intra-assay coefficient of variation (CV) was 2.0%. For intermediate precision, the inter-assay CV was lower in CLEIA (7.1%) than Luminex (10.7%, *p* = 0.009) and ELISA (10.8%, *p* = 0.009), primarily due to improved intermediate precision in the higher CSF Aβ42 concentrations. We conclude that the automated CLEIA generated reproducible CSF Aβ42 measures with improved intermediate precision over experienced operators using Luminex assays and ELISA, and are highly correlated with the manual Aβ42 measures.

## Introduction

Cerebrospinal fluid (CSF) levels of proteins and peptides associated with neuritic plaques and neurofibrillary tangles can enhance the accurate etiologic diagnosis of Alzheimer’s disease (AD) (Shaw et al., 2009; Jack et al., 2010, 2018). These markers –beta-amyloid peptides (Aβ38, Aβ40, Aβ42), (Adamczuk et al., 2015; Olsson et al., 2016; Howell et al., 2017) total and phosphorylated tau (Arai et al., 2000; Hampel et al., 2004; Fagan et al., 2009) – are measured in research and commercial laboratories around the world, but there remain key obstacles in their broader application. These include the need to purchase a research-based assay platform, pre-analytical and analytical variability, (Fourier et al., 2015; Leitao et al., 2015) and the need for experienced operators. While international quality control programs (Toledo et al., 2012; Kang et al., 2013; Pannee et al., 2016) aim to optimize measurement variability across assays, reagents, platforms, standards, operators, and algorithms, technological solutions including process engineering and assay automation can potentially reduce variability introduced by human operators that influence assay performance.

Immunoassays targeting Aβ42, t-Tau, and p-Tau_181_ have predominated the landscape of CSF AD biomarker analysis to date, although mass spectrometry-based assays are under development (Pannee et al., 2016). Compared to solid-phase enzyme linked immunosorbent assays (ELISA) and fluid-phase Luminex assays which require manual reagent addition and removal on multi-well plates, automated analyzers are proposed to have better repeatability (variability within the same assay) and intermediate precision (variability between different assays). The Fujirebio Lumipulse^®^ system and the Roche Elecsys^®^ have shown consistent inter-assay measures in the serum, with coefficient of variation (CV) in the range of 1.2–10% for hepatitis B antigens, (Yang et al., 2016) tumor markers, (Marlet and Bernard, 2016) cortisol, (Vogeser et al., 2017) and interleukin-13 (Palme et al., 2017). In the CSF, a recent multi-center study using synthetic Aβ42 peptides in artificial CSF reported inter-assay CV of <5% on the Elecys^®^ system, (Bittner et al., 2016) but the intermediate precision of endogenous Aβ42 in human-derived CSF samples in these automated analyzers remains unknown. Because most cohort, (Ellis et al., 2009; Shaw et al., 2009; Bendlin et al., 2012) clinicopathologic, (Roher et al., 2009; Hu et al., 2010; Li et al., 2015) and pharmacological (Blennow et al., 2012; Kennedy et al., 2016) studies to-date have relied on one of the non-automated assays, it is also important to determine the measurement correlation between the three assay formats. Here we selected 30 human CSF samples representing a range of physiologic Aβ42 levels, characterized the correlation between CSF Aβ42 measurements from different assay types, and assessed the repeatability and intermediate precision of each assay.

## Materials and Methods

### Standard Protocol Approvals and Patient Consents

This study was carried out in accordance to US Code of Federal Regulations Title 45 Part 46 Protection of Human Subjects, and Emory University and Emory School of Medicine policies. The protocols were approved by the Emory University Institutional Review Board. Banked CSF samples were used for this study, and all subjects had previously given written informed consent according to the Declaration of Helsinki for long-term sample storage and future analysis.

### CSF Pooling and Aliquotting

Cerebrospinal fluid samples were all previously collected using a modified AD Neuroimaging Initiative protocol (Hu et al., 2013). Briefly, CSF was collected into 15 mL polypropylene tubes via a 24-gauge atraumatic needle and syringe aspiration without overnight fasting. Polypropylene tubes were inverted several times, and CSF was aliquotted (500 μL), labeled, and frozen at -80°C until analysis.

Cerebrospinal fluid samples from 30 subjects were selected for the study (**Table 1**). Subjects were chosen to represent a wide range of Aβ42 concentrations (previously measured using Luminex): nine had normal cognition, 12 had mild cognitive impairment, six had AD dementia, and three had other non-AD dementias (one each for corticobasal syndrome, dementia with Lewy bodies, and progressive supranuclear palsy).

**Table 1:** Characteristics of subjects and assays according to CSF Aβ42 concentrations.

	Low(*n* = 10)	Intermediate(*n* = 10)	High(*n* = 10)
Female (%)	3 (30%)	7 (70%)	3 (30%)
Age, yr *(SD)*	72.7 (7.9)	65.5 (8.5)	72.5 (8.4)
Diagnosis			
Normal cognition	3	2	4
Mild cognitive impairment	4	2	6
AD dementia	2	4	0
Other dementia	1	2	0
Aβ42_CLEIA_, pg/mL *(SD)*	219.5 (50.2)	327.7 (51.0)	821.5 (194.4)
Aβ42_Luminex_, pg/mL *(SD)*	105.7 (29.3)	173.3 (42.4)	400.9 (68.6)
Aβ42_ELISA,_ pg/mL *(SD)*	265.9 (56.6)	363.3 (66.1)	698.0 (97.6)
t-Tau_Luminex_, pg/mL *(SD)*	51.2 (50.9)	64.3 (62.1)	33.5 (7.8)
p-Tau_Luminex_, pg/mL *(SD)*	22.9 (24.7)	42.2 (48.8)	7.1 (6.9)
Intra-assay CV (repeatability)			
CLEIA	2.4%	2.1%	1.4%
Luminex	12.0%	13.3%	9.4%
ELISA	3.3%	3.6%	3.1%
Inter-assay CV (intermediate precision)			
CLEIA	10.1%	7.1%	4.0%
Luminex	11.8%	11.5%	8.8%
ELISA	14.6%	9.8%	7.8%

**FIGURE 1:**
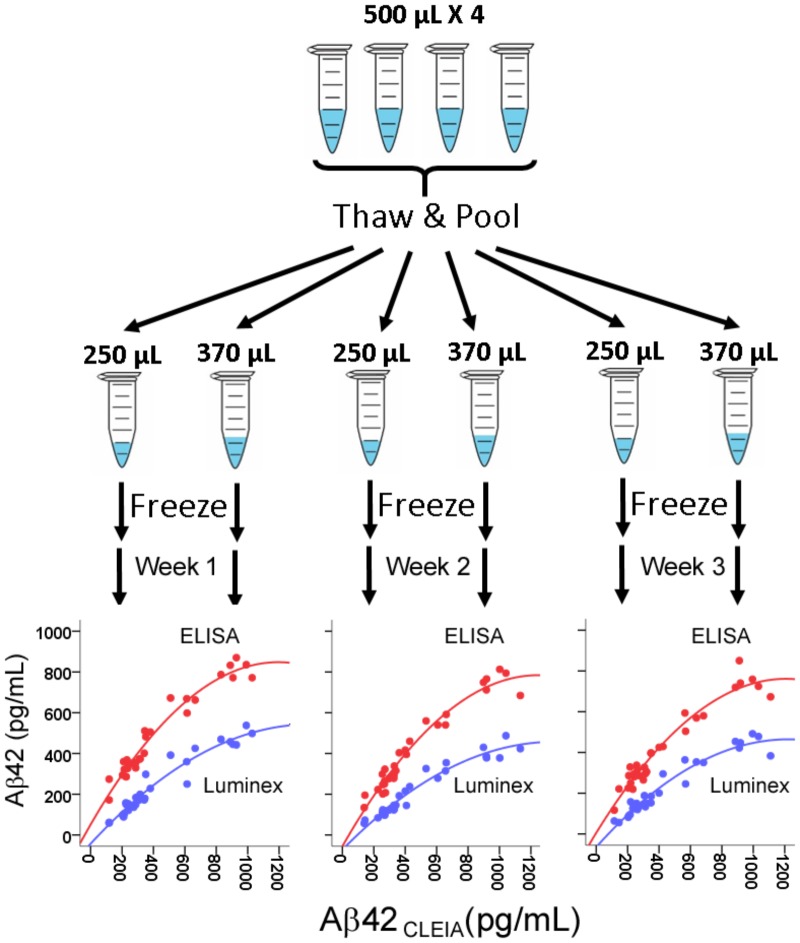
Assessment of correlation between CSF Aβ42 levels measured in CLEIA, Luminex, and ELISA across three weekly runs. For each subject, four frozen 500 μL aliquots were thawed, pooled, and realiquoted. On the first day of each week, aliquots were thawed for analysis on the three platforms. In the correlational figures, CLEIA measures are presented on the *X*-axis, and the ELISA and Luminex measures are presented on the *Y*-axis.

Because we wished to compare the platforms’ performance over three weekly runs, we first generated identical CSF aliquots for all runs (**Figure 1**). For each subjects, four 500 μL CSF aliquots were thawed at room temperature, vortexed, and pooled into a 5 mL polypropylene tube. The pooled 2 mL aliquot was then vortexed and separated into three 250 μL aliquots and three 370 μL aliquots. All aliquots were then re-frozen to ensure the same freeze-thaw cycles in addition to the same number of tube transfers.

### CSF Analysis

On the first day of each study week, every sample was analyzed in triplicates (three wells) on the automated Lumipulse^®^ Aβ42 chemiluminescent enzyme immunoassay (CLEIA), INNO-Bia Alzbio3 Luminex assay, and INNOTEST^®^ Aβ42 ELISA. In the morning, one 370 μL aliquot was thawed at room temperature for each subject and analyzed using the ELISA according to the manufacturer’s protocol on two separate 96-well plates. A full set of kit standards and two kit controls were included in each plate. The inter-plate CV for the kit controls were 4.7 and 18.0% on Week 1, 2.9, and 4.8% on Week 2, and 21.6 and 13.4% on Week 3. The remaining CSF from each 370 μL aliquot was transferred to the CLEIA sample cups for Aβ42 analysis following the manufacturer’s protocol.

In the afternoon, one 250 μL aliquot was thawed at room temperature for Luminex assays according to a modified manufacturer’s protocol: all samples were vortexed vigorously for exactly 15 s immediately prior to plate loading, (Hu et al., 2015) and the bead count (performed the next day) was reduced to 75 to minimize fluorescence loss. As with the ELISA, samples were divided between two plates, with a full set of kit standards and two kit controls on each plate. The inter-plate CVs for the two kit controls were 0.2 and 0.9% on Week 1, 6.1 and 2.8% on Week 2, and 4.7 and 1.9% on Week 3.

### Statistical Analysis

Statistical analyses were performed using IBM SPSS version 24.0 (Armonk, NY, United States). Aβ42 concentrations measured by CLEIA, Luminex, and ELISA were all log-transformed prior to Pearson’s correlation analysis due to their non-normal distribution, with *p* < 0.01 to account for multiple comparisons. Repeatability for each assay was assessed by averaging CV for the triplicate concentration values within the same run, and intermediate precision for each assay was assessed by calculating CV for the three weekly concentrations. Analysis of variance (ANOVA) and analysis of co-variance (ANCOVA) were used to determine whether intermediate precision differed between the three assays before and after adjusting for Log_10_-transformed concentrations.

### Data Availability

The raw data supporting the conclusions of this manuscript will be made available by the authors, without undue reservation, to any qualified researcher.

## Results

### Aβ42 Levels Were Highly Correlated Between the Three Assays

There was a very high degree of correlation between concentrations from all three platforms (*R* = 0.964–0.973 between CLEIA and Luminex, 0.961–0.967 between CLEIA and ELISA, and 0.932–0.969 between Luminex and ELISA). Regression analysis showed that a quadratic – rather than linear – relationship better converted measures from ELISA and Luminex to CLEIA due to relatively higher CLEIA measures than ELISA/Luminex measures in the high concentration range [**Figure 1**, Aβ42_CLEIA_ = 63.32 + 0.32Aβ42_ELISA_ + 0.001Aβ42_ELISA^2^_, Aβ42_CLEIA_ = 100 + 0.92Aβ42_Luminex_ + 0.002Aβ42_Luminex^2^_]. In contrast, the relationship between Luminex and ELISA was linear in all concentrations (Aβ42_Luminex_ = 0.66Aβ42_ELISA_ -66.35).

### Repeatability

The average intra-assay CV for Aβ42_CLEIA_ was <2.5% for all Aβ42 levels (**Table 1**). These were comparable to values for Aβ42_ELISA_, but lower than values for Aβ42_Luminex_ as expected from the reduced bead count per well. Among 90 triplicates, two samples (2%, from the same subject) on CLEIA and five samples from ELISA had intra-assay CV greater than 5%.

### Intermediate Precision

**FIGURE 2:**
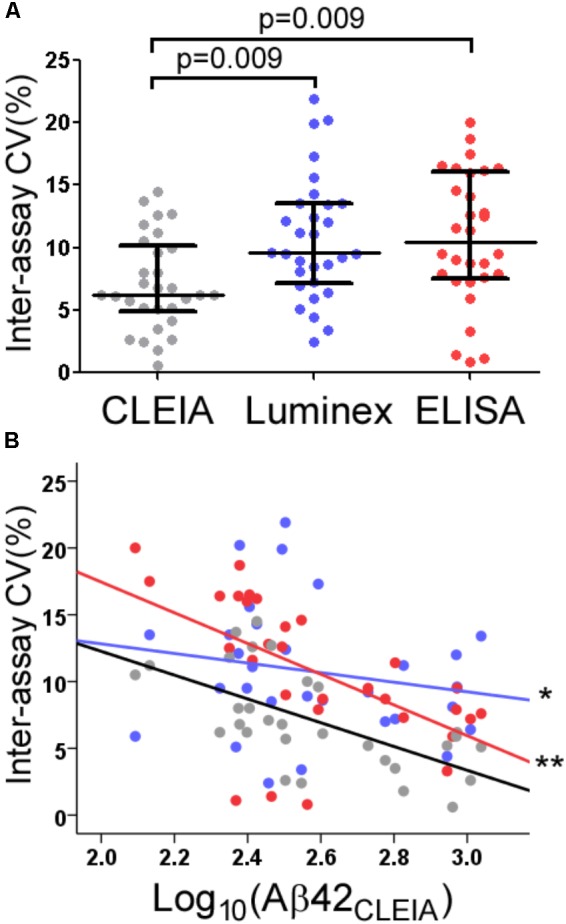
Intermediate precision of CLEIA-, Luminex-, and ELISA-based CSF Aβ42 measures. Across all concentrations, Aβ42_CLEIA_ measures had lower CV than Aβ42_Luminex_ or Aβ42_ELISA_ **(A)**. Further examination of the differences in CV **(B)** showed that Aβ42_CLEIA_ had lower inter-assay CV than Aβ42_Luminex_ at higher concentrations (^∗^), and Aβ42_ELISA_ at all concentrations (^∗∗^).

The average inter-assay (within laboratory) CV for Aβ42_CLEIA_ ranged from 4.0% for high concentrations to 10.1% for low concentrations (**Table 1**). When all concentrations were analyzed together, ANOVA showed that Aβ42_CLEIA_ (7.1%) had lower inter-assay CV than the other platforms [**Figure 2A**, 10.7% for Aβ42_Luminex_ (*p* = 0.0090), 10.8% for Aβ42_ELISA_ (*p* = 0.009)]. ANCOVA adjusting for Aβ42_CLEIA_ concentrations (**Figure 2B**) showed that, in addition to an inverse relationship between CV and concentration (*p* < 0.001), CLEIA had greater intermediate precision than Luminex at higher concentrations (*p* = 0.001 for assay X concentration), and greater intermediate precision over ELISA at all concentrations (*p* < 0.001 for assay).

## Discussion

While CSF biomarkers have shown great promise in the ante-mortem prediction of AD pathology, pre-analytical and analytical processes need to be standardized and simplified for inclusion into existing clinical workflows. Because every step – pre-analytical or analytical – affords an opportunity for within-operator and between-operator variability, automation of the analytical portion of Aβ42 measurements likely reduced the within-operator, inter-assay imprecision (Bittner et al., 2016; Chiasserini et al., 2016). Operator-associated imprecision in the busy clinical laboratories is likely much greater, as published inter-assay CV values generally come from experienced biomarker laboratories (5.3–14% for Luminex and 6.4–25% for ELISA) with experienced staff dedicated to these specialized assays (Kang et al., 2013). At the same time, inconsistent reporting of intermediate precision – particularly related to source material [e.g., synthetic peptides, (Bittner et al., 2016) pooled CSF (Chiasserini et al., 2016)] – prevents comparison across manual and automated platforms, as well as across different automated platforms. While the study design here required a modest amount of CSF from each subject, involving banked samples from multiple centers or recruiting study volunteers specifically for the purpose of assay standardization can streamline future studies for new assays or analyzers.

The strong correlation between Aβ42_CLEIA_ and the other Aβ42 measures also permits comparison between legacy and new data. The correlation observed here is stronger than that reported between another automated analyzer and the same ELISA we used (Chiasserini et al., 2016). The difference likely resulted from our use of CLEIA and ELISA from the same manufacturer. Our observation of non-linear relationship in higher Aβ42 concentrations also warrants follow-up, as this observation was not specifically examined with the other analyzers. Nevertheless, we report good repeatability, intermediate precision, and strong correlation with established Aβ42 assays. Because the automated analyzer already has FDA clearance in the United States, CSF Aβ42 measurement will likely not be limited to specialized centers in the near future. At the same time, because CSF collection and handling are still manual in nature, there is an ever more urgent need to standardize these pre-analytical steps through processing engineering and possibly automation.

## Author Contributions

AK, JH, and WH contributed conception and design of the study. AK and JH organized the dataset. AK and WH performed the statistical analysis. AK, JH, and WH wrote sections of the manuscript, contributed to the revision of the manuscript, read and approved the submitted version.

## Conflict of Interest Statement

WH has a patent on the use of CSF biomarkers to diagnose FTLD-TDP (US 9,618,522); has received research support from Avid Radiopharmaceuticals (Philadelphia, PA, United States) and Fujirebio US (Malvern, PA, United States); serves as a consultant to AARP, Inc., Locks Law Firm, Interpleader Law, and ViveBio LLC; has received travel support from Hoffman-LaRoche and Abbvie. The remaining authors declare that the research was conducted in the absence of any commercial or financial relationships that could be construed as a potential conflict of interest.
